# The Design of a Plant-Based Biopesticide Formulation with Extracts and Derivatives Containing Naphthoquinones

**DOI:** 10.3390/plants14223427

**Published:** 2025-11-09

**Authors:** María Isabel Calvo

**Affiliations:** 1 Department of Pharmaceutical Sciences, School of Pharmacy and Nutrition, University of Navarra, Irunlarrea 1, 31008 Pamplona, Spain; mcalvo@unav.es; 2 IDISNA-Instituto de Investigación Biosanitaria de Navarra, 31008 Pamplona, Spain

**Keywords:** *Alternaria alternata*, *Botrytis cinerea*, *Penicillium expansum*, *Rhizopus stolonifer*, mould rot, antimicrobial

## Abstract

This study aimed to determine the antifungal activity of various compounds and develop a novel antifungal formulation against fungal pathogens, including *Alternaria alternata*, *Botrytis cinerea*, *Penicillium expansum*, and *Rhizopus stolonifer.* A total of 32 plant-derived secondary metabolites and three extracts (dichloromethane, ethyl acetate, and methanol) from *Lawsonia inermis*, *Juglans regia*, and *Drosera intermedia* were screened at a concentration of 250 ppm. The chemical composition of the *D. intermedia* ethyl acetate extract was characterized using chromatographic techniques. Subsequently, an emulsifiable concentrate formulation from this extract was prepared, and its efficacy was evaluated at concentrations ranging from 250 to 2000 ppm. The *D. intermedia* ethyl acetate extract was found to contain three flavonoids (1.4%) and three naphthoquinones (2.8%). The formulation exhibited optimal effect at 1000 ppm. Overall, the high efficacy of the formulation containing the dried *D. intermedia* extract (10:1, ethyl acetate) positions it as a promising and viable alternative to synthetic fungicides.

## 1. Introduction

Postharvest diseases are one of the major problems for the fruit industry, with particular emphasis on those caused by pathogenic filamentous fungi that result in rots. To facilitate preservation and prevent significant economic losses, antifungal agents are often applied during fruit processing and storage. Postharvest diseases are classified into two main categories based on the timing of infection.

Infections are caused by latent pathogens that infect the fruit in the crop but remain dormant until after harvest, when they cause rot development. This category includes “grey mould rot” (*Botrytis cinerea*), “brown mould rot” (*Phytophthora citrophthora*), “core rot” (*Alternaria alternata*), anthracnose (*Colletotrichum gloesporioides*), and peduncular rot caused by *Lasiodiplodia theobromae* and *Phomopsis citri*.

Wound pathogens, on the other hand, contaminate the fruit through injuries sustained during harvesting and subsequent handling. This group includes “green mould rot” (*Penicillium digitatum*), “blue mould rot” (*Penicillium expansum*), “sour rot” (*Geotrichum citri-aurantii*), and “*Rhizopus* rot” caused by the genus *Rhizopus* (*Rhizopus stolonifer*) [1].

The global food supply chain is under constant pressure, primarily due to significant food loss and waste that occurs from production to consumption. According to the Food and Agriculture Organization of the United Nations (FAO), about one-third of the food produced globally is lost or wasted annually, fruits, vegetables, roots, and tubers being particularly affected, reaching losses up to 45% [2].

This issue is a central focus of the global sustainability agenda, and it is explicitly addressed in Goal 12.3 of the UN 2030 Agenda for Sustainable Development, which calls for halving per capita food waste and substantially reducing food losses across the entire supply chain, including at the postharvest level [3].

Historically, the main method for mitigating these losses has been the use of synthetic chemical fungicides applied after harvest [4]. These compounds have proven highly effective, reducing fungal losses to a range of 2–4%, a stark contrast to the 15–30% losses that occur in the absence of treatment or with refrigeration alone [5]. However, the short-term effectiveness of these fungicides is overshadowed by their serious long-term drawbacks. The continuous application of these chemicals raises increasing concerns about the accumulation of toxic residues in food and the environment, posing a risk to human health and requiring complex waste treatments [6]. Furthermore, prolonged use has led to the development of pathogen resistance, making conventional treatments increasingly ineffective and unsustainable [7].

This scenario has driven the demand for research and development of alternative and sustainable biocides. The next generation of postharvest treatments must prioritize safety and efficacy by using compounds with a low toxicological profile for humans and animals and a minimal environmental residual impact. Consequently, attention has shifted toward GRAS (“Generally Recognized as Safe”) substances and, more prominently, natural extracts from plants, microorganisms, and animals [8]. These natural compounds offer a safer solution and can be applied in various ways, directly or as part of protective coatings, representing a promising path towards a safer and more environmentally conscious food industry [9].

Previous studies have demonstrated that plant extracts from medicinal plants are a promising alternative for preventing postharvest rot [10]. Their efficacy is attributed to their low toxicity and proven antibacterial and antifungal activity in both in vitro and in vivo assays. This biological activity is due to their rich content of secondary metabolites, including polyphenols, flavonoids, quinones, tannins, terpenes, alkaloids, saponins, sterols, phenylpropanoids, glycosinolates, and isothiocyanates [11,12].

The primary aim of this study was to develop alternative and sustainable postharvest antifungal treatments to replace conventional fungicides, thereby extending the shelf life and ensuring the quality of fruits. This work contributes directly to several UN 2030 Sustainable Development Goals (SDGs), particularly SDG 2 (Zero Hunger) by reducing postharvest food loss and SDG 3 (Good Health and Well-being) by promoting safer food systems with natural, non-toxic alternatives to synthetic chemicals. To achieve these sustainable objectives, the research focused on the following stages: (i) screening of natural secondary metabolites for antifungal activity against *Alternaria alternata*, *Botrytis cinerea*, *Penicillium expansum*, and *Rhizopus stolonifer*; (ii) searching for plant extracts and derivatives from medicinal plants with high antifungal activity and favourable environmental profile; (iii) designing a sustainable formulation for postharvest application that is effective, safe, and environmentally friendly.

## 2. Results and Discussion

The global biopesticide market is undergoing significant expansion, with Europe alone representing 45% of the total demand. This growth is a direct response to the phasing out of many synthetic pesticides, which have either been banned or are under strict regulatory review due to their environmental impact, the emergence of pathogen resistance, and associated health concerns. Consequently, the search for non-chemical, natural alternatives has become a research priority. These compounds are particularly attractive because of their inherent efficacy, straightforward extraction methods, safety for both the environment and human health, and rapid degradation. Recent studies have specifically focused on non-chemical and biological control techniques for managing postharvest diseases leading to the development of highly effective antifungal agents, achieved through the comprehensive screening of natural products and the optimization of their active molecular structures [13]. As a result, several natural fungicides, such as thyme oil, cinnamon oil, and citrus seed extract, are now commercially available [14].

### 2.1. Antifungal Activity of Plant-Derived Secondary Metabolites

Plant secondary metabolites, particularly phenolic compounds like phenolic acids, coumarins, flavonoids, and quinones, show significant potential as antibacterial and antifungal agents. The remarkable antifungal efficacy of polyphenols is due to a wide range of interconnected mechanisms that allow them to act as potent agents against fungal pathogens. Their efficacy lies not in a single pathway, but in their ability to simultaneously attack multiple cellular targets, thereby compromising fungal viability and survival. Their primary mechanisms of action often involve cell membrane interaction, enzymatic inhibition, oxidative stress generation, and metal ion chelation. These multifaceted effects make them potent agents against fungal pathogens [15,16].

The antifungal efficacy, expressed as a percentage of growth inhibition, of 32 phenolic standards (1–32), classified by their phytochemical families, was studied against four pathogenic fungal species: *Alternaria alternata*, *Botrytis cinerea*, *Penicillium expansum*, and *Rhizopus stolonifer* (Table 1).

**Figure 1:**
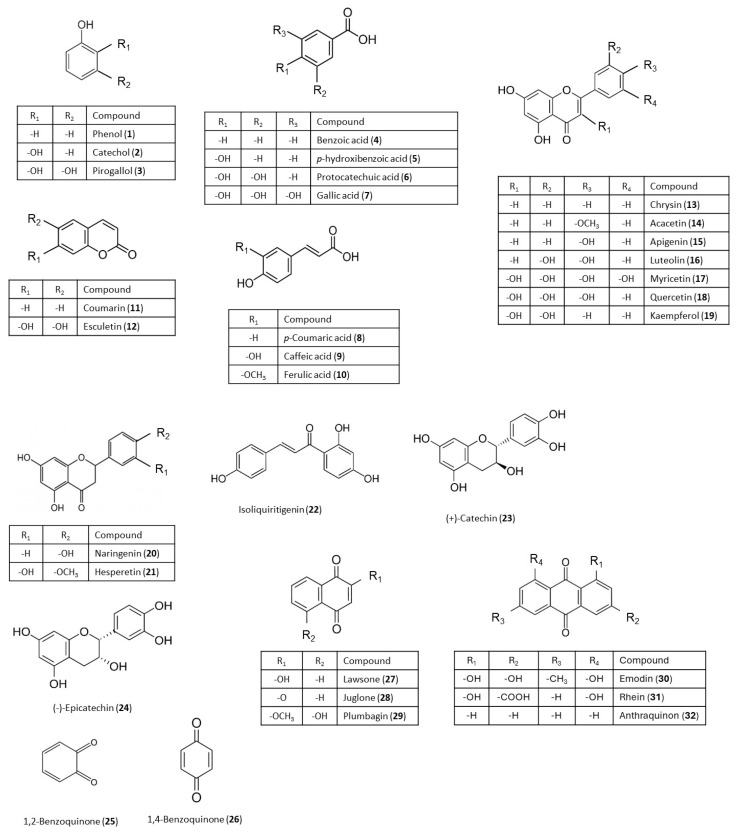
Chemical structures of secondary metabolites.

The chemical structures of the secondary metabolites tested are shown in Figure 1.

Simple phenols are compounds with at least one hydroxyl (-OH) group directly attached to an aromatic ring. Among the ones studied, a clear difference in activity was observed. Pyrogallol (**3**), a trihydroxy phenol, showed notable efficacy against *A. alternata* (71%) and *B. cinerea* (66%) aligning with the high potency suggested for polyphenols with vicinal -OH groups due to their pro-oxidant capacity. However, its efficacy dropped drastically against *P. expansum* and *R. stolonifer*, indicating a specificity in the mode of action. Catechol (**2**), a dihydroxy phenol, also showed activity against *B. cinerea* (29%) and *P. expansum* (19%), while the basic phenol (**1**), with a single -OH group, was the least active. The results confirm that each additional hydroxyl group not only increases polarity but also enhances the ability to form hydrogen bonds, which is fundamental for interaction with fundamental targets. The position and number of -OH groups are important, as arrangements like those in catechol (ortho-) and pyrogallol (*ortho*- and *para*-) favour redox cycles and the generation of reactive oxygen species (ROS), causing oxidative damage and cellular lysis. The antifungal activity is often associated with fungal cell membrane destabilization and enzymatic inhibition [15].

Phenolic acids, aromatic compounds with a carboxylic side chain, are divided into hydroxybenzoic acids and hydroxycinnamic acids. Hydroxybenzoic acids are characterized by a short side chain (-COOH) directly attached to an aromatic ring and one or more -OH groups. Of the four acids evaluated—benzoic acid (**4**), *p*-hydroxybenzoic acid (**5**), protocatechuic acid (**6**), and gallic acid (**7**)—low activity was observed for the first two. Protocatechuic acid (**6**), with two vicinal -OH groups, showed activity against *P. expansum* (41%) and *B. cinerea* (28%), while gallic acid (**7**), with three vicinal hydroxyls, exhibited high activity against *B. cinerea* (57%) and *R. stolonifer* (62%), although it showed less activity against *A. alternata* (12.1%) and *P. expansum* (0.4%) [17]. Hydroxycinnamic acids, which have an unsaturated three-carbon side chain (-CH=CH-COOH), showed more moderate activity. Only caffeic acid (**9**) and ferulic acid (**10**) showed activity against *A. alternata* (25% vs. 21%), while *p*-coumaric (**8**) acid was inactive. The antifungal activity of phenolic acids, in general, is conditioned by the number and position of hydroxyl groups and their carboxylic side chain. The acidity and polarity of these compounds directly influence their ionization and ability to cross fungal membranes. Weakly acidic molecules like phenolic acids, for example, become protonated in acidic external environments, facilitating their passive diffusion across the cell membrane. Once inside the more alkaline fungal cell, they deprotonate and become trapped, causing cytoplasmic acidification and metabolic disruption [18,19]. The activity of hydroxycinnamic acids has been previously described: *p* -coumaric acid, for example, can act as an uncoupler of oxidative phosphorylation in *Botrytis cinerea* [20], while caffeic acid and ferulic acid also possess antimicrobial activity linked to membrane disruption and enzymatic inhibition [21,22].

Coumarins, natural lactones derived from benzopyran-2-one, are known for their antifungal properties [23]. Their action is attributed to their ability to form non-covalent interactions with various active sites in living organisms [24]. Unsubstituted coumarin (**11**) showed no antifungal activity, whereas esculetin (6,7-dihydroxycoumarin, **12**) displayed low activity against *B. cinerea* (7%) and *P. expansum* (10%). These results suggest that while the basic benzopyran-2-one skeleton provides lipophilicity, hydroxylation at the C6 and C7 positions may enhance antifungal activity. This finding is consistent with other authors’ who demonstrated that coumarins with simple structures possess weak to moderate antifungal activity against *A. fumigatus* and *A. flavus* [25].

Flavonoids, a diverse class of plant polyphenols, possess a basic C6-C3-C6 structure, composed of two benzene rings (A and B) connected by a three-carbon chain that typically forms a heterocycle (C ring). This structural backbone gives rise to several subclasses (such as flavones, flavonols, and chalcones), whose specific features modulate their biological activity, including antifungal properties against a wide range of pathogenic organisms [26]. In this study, all tested flavonoids were active against *B. cinerea* and *R. stolonifer* but inactive against *A. alternata* and *P. expansum*, though with variable results. Flavones, with a C2-C3 double bond and a carbonyl at C4, have a planar structure that favours intercalation into fungal DNA or specific interactions with membrane proteins. Apigenin (**15**) and luteolin (**16**) showed high inhibition percentages against *B. cinerea* (28%), and against *R. stolonifer* (19%). In contrast, chrysin (**13**) and acacetin (**14**), which lack hydroxyl groups on the B ring, showed no antifungal activity. These results support the idea that a greater number of hydroxyl groups leads to higher activity.

Flavonols are like flavones but with an additional hydroxyl group at C3. This type of structure can sequester essential metal ions for fungal enzymes or generate ROS through redox cycles. The results show that quercetin (**18**) and myricetin (**17**) are active against *R. stolonifer* (33% vs. 16%), while kaempferol (**19**), which only has a C3-OH, did not show antifungal activity.

Flavanones have a saturated C ring, resulting in a less planar structure. Despite this, naringin (**20**) was active against *B. cinerea* (26%) and hesperetin (**21**) against *R. stolonifer* (29%).

Meanwhile, chalcones are biosynthetic precursors to cyclic flavonoids and lack a closed C ring. Their open structure and highly conjugated three-carbon chain provide them with high electrophilic reactivity. Isoliquiritigenin (**22**) was active against *A. alternata* (39%) and *P. expansum* (25%), a notable finding that contrasts with the inactivity of the other tested flavonoids. Their antimicrobial efficacy is largely attributed to their interaction with the cell membranes, binding proteins, and cell walls of target pathogens [27].

Catechins (catechin (**23**) and epicatechin (**24**)) are flavan-3-ols, which are characterized by a saturated C-ring and a hydroxyl group at the C3 position. Their non-planar structure and multiple hydroxyls contribute to their ability to disrupt the fungal membrane, inhibit enzymes, and generate ROS. Despite existing literature confirming their broad spectrum of activity [28], they showed low activity in this study, suggesting a lack of specificity against the tested strains.

Quinones constitute a large class of compounds with a cyclic, α,β-unsaturated diketone. They are classified into three main types: benzoquinones (with a single quinone ring), naphthoquinones (two fused rings, one of which is a quinone), and anthraquinones (three fused rings, one of which is a quinone).

The tested benzoquinones, 1,2-benzoquinone (**25**) and 1,4-benzoquinone (**26**), showed antifungal activity. 1,4-benzoquinone exhibited higher activity against all four fungi evaluated: *A. alternata* (25%), *B. cinerea* (15.2%), *P. expansum* (28%), and *R. stolonifer* (30%). These results suggest that the *para* position of the keto groups is important for its activity spectrum [29].

Among all compounds tested, the naphthoquinones generally showed the highest and broadest antifungal activityindicating a broad spectrum of action. Specifically, lawsone (**27**) and plumbagin (**29**) exhibited a maximum inhibition percentage ranging from 64% to 86%, indicating a broad spectrum of action. Plumbagin (**29**), with its methyl and hydroxyl groups on the ring, exhibited the highest inhibition percentages of all evaluated compounds (*A. alternata*: 80%; *B. cinerea*: 65%; *P. expansum*: 85%; *R. stolonifer*: 86%). Lawsone (**27**), with an -OH group at position 2, also presented high inhibition percentages (*A. alternata*: 77%; *B. cinerea*: 86%; *P. expansum*: 64%; *R. stolonifer*: 80%). Juglone (**28**), on the other hand, showed antifungal activity, though lower than lawsone and plumbagin, standing out particularly against *A. alternata* (31%) and *R. stolonifer* (50%). These results suggest that the presence, position, and nature of substituents on the naphthoquinone system are important for its activity [30]. The high activity of plumbagin (**29**) aligns with previous studies establishing the structure-activity relationship in naphthoquinones [31]. For instance, a hydroxyl at position 5 adjacent to the carbonyl allows chelate formation, increasing antifungal activity, while electron-donating groups at positions 2 and 3 of the 1,4-naphthoquinone ring, such as -CH_3_ and -OCH_3_, can improve binding affinity [32].

The anthraquinones, emodin (**30**) and rhein (**31**), also presented activity against *A. alternata* (21% and 36%) and *B. cinerea* (49% and 47%), though the unsubstituted anthraquinone (**32**) showed lower values (11%). These compounds are known for their antimicrobial potential, affecting the cell membrane and fungal respiration [33,34].

Overall, these results offer a comprehensive understanding of the structure-activity relationship of polyphenols, underscoring that the number and position of hydroxyl groups, along with the presence of the other substituents, are fundamental for their antifungal activity.

**Figure 2:**
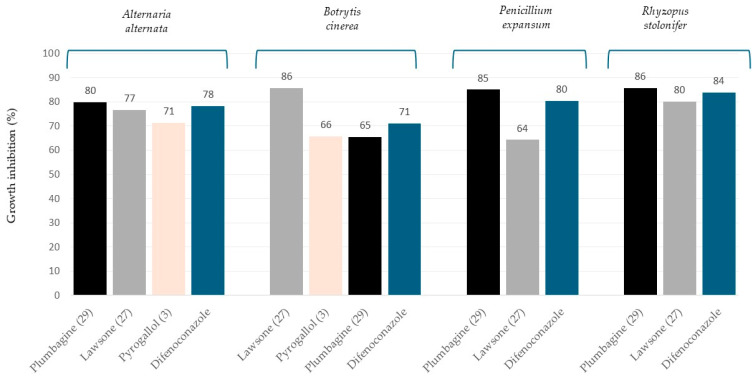
TOP 10 ranking of antifungal compounds expressed as growth inhibition percentage (%) values.

Based on their growth inhibition percentage, the top 10 antifungal compounds are shown in Figure 2.

While the reference fungicide difenoconazole provided consistent and high inhibition, it was not the most effective compound across all tested species. Plumbagin (**29**) demonstrated a clear advantage, outperforming difenoconazole against *P. expansum* (85% vs. 80%) and *R. stolonifer* (86% vs. 84%). This superior performance highlights plumbagin as a prime candidate for the development of new biofungicide. Likewise, lawsone (**27**) showed notable activity against *B. cinerea* (86%), exceeding that of difenoconazole and making it a compound of significant interest for controlling grey mould. Although compounds like pyrogallol (**3**) exhibited moderate antifungal activity against *A. alternata* (71%) and *B. cinerea* (66%), these initial findings strongly suggest that naphthoquinones represent the most promising class for continued research.

### 2.2. Antifungal Activity of Plant Extracts with Naphthoquinones

Naphthoquinones occur as yellow or orange pigments, which are products of secondary metabolism in higher plants from specific families, including *Bignoniaceae*, *Droseraceae*, *Juglandaceae*, *Plumbaginaceae*, and *Lythraceae*. These compounds are of significant scientific interest due to their diverse pharmacological activities, including antimicrobial, antifungal, antiparasitic, insecticidal, anti-inflammatory, antioxidant, and cytotoxic activities [30,32,35,36,37].

To identify a plant extract with antifungal activity against the four tested fungi, three medicinal plants containing naphthoquinones were selected: *Lawsonia inermis*, *Juglans regia*, and *Drosera intermedia*. *Lawsonia inermis* Linn (*Lythraceae*), commonly known as henna, is a rich source of bioactive compounds, with lawsone being one of the most important [38]. It has a broad spectrum of pharmacological activities, including antioxidant, anti-inflammatory, analgesic, antiparasitic, hepatoprotective, antifungal, antitumor, wound-healing, and hypoglycaemic effects [39,40]. *Juglans regia* L. (*Junglaceae*), or the walnut tree, is widely distributed globally and contains over 200 chemical compounds. Naphthoquinones constitute 70% of its quinone content, with juglone being one of the major ones. Pharmacological studies have showed that juglone possesses excellent antitumor, antioxidant, and antibacterial properties [41]. *Drosera intermedia* Hayne (*Droseraceae*) is a carnivorous plant species used as a medicinal plant, and its efficacy is primarily attributed to its content of naphthoquinones and flavonoids [42].

Table 2 shows the extraction yields of the three species using the three solvents (dichloromethane, methanol, and water), as well as the growth inhibition percentages against the four tested fungi.

The results indicate that the extraction solvent is an important factor influencing both the chemical composition and the antifungal efficacy of the extracts. In all three cases, the ethyl acetate extract showed the highest activity, despite the methanol extracts having a higher extraction yield.

The dichloromethane extract of *L. inermis* showed no antifungal activity. In contrast, the ethyl acetate extract, with a yield of 8.51%, was the most effective, with its activity against *P. expansum* (95%) even surpassing that of the lawsone standard (64%) and its activity against *A. alternata* reaching 71%. Despite having the highest yield (20%), the methanol extract exhibited significantly lower activity.

The ethyl acetate extract of *J. regia* was the most active, with 84% inhibition against *R. stolonifer*, significantly outperforming to juglone standard (50%). The extract was also more effective against *A. alternata* (38%) than the juglone standard (31%). As well as *L. inermis*, the methanol extract had a higher yield (13%) but lower activity.

The same pattern was observed with the *D. intermedia* extracts, where the ethyl acetate extract showed higher activity than the plumbagin standard in three of the four tested fungi: 87% against *A. alternata*, 96% against *B. cinerea*, and 85% against *R. stolonifer*.

These results align with the literature, which has also reported the efficacy of ethyl acetate extracts from these plants. Specifically, *L. inermis* ethyl acetate extract has demonstrated activity against a wide range of fungi, including *Candida* sp., *Cryptococcus* sp., *Aspergillus* sp., *Penicillium* sp., *Tinea* sp., and *Blastomyces* sp. [43], as well as *Pseudomonas aeruginosa* [44]. Similarly, *J. regia* extracts have shown antibacterial effects against various pathogenic microorganisms. Prior studies have highlighted the superior antibacterial activity of its ethyl acetate extracts against *Escherichia coli* and *Bacillus cereus* [45], in addition to their effectiveness against *C. albicans* [46].

Very few studies have addressed the antifungal activity of *D. intermedia*. However, some authors have reported that the most nonpolar extract was effective against *Aspergillus fumigatus, A. niger*, and *A. flavus*, with activities comparable to miconazole. The efficacy of this extract is attributed to its major compound, plumbagin. This same extract has also been shown to exhibit antibacterial activity against *Staphylococcus epidermidis* and *Candida albicans* [42,47].

In this regard, these results are promising because they demonstrate that using plant extracts instead of isolated pure compounds offers significant advantages. The synergy among the extract’s various components can enhance antifungal efficacy. This approach also provides benefits such as a more favourable safety profile, improved bioavailability, and multi-target action on biological pathways. Furthermore, from an economic standpoint, obtaining plant extracts is a simpler, more cost-effective, and environmentally friendly process compared to the complex and expensive synthesis or isolation of pure compounds [48,49,50].

### 2.3. Characterization of the Drosera intermedia Ethyl Acetate Extract

**Figure 3:**
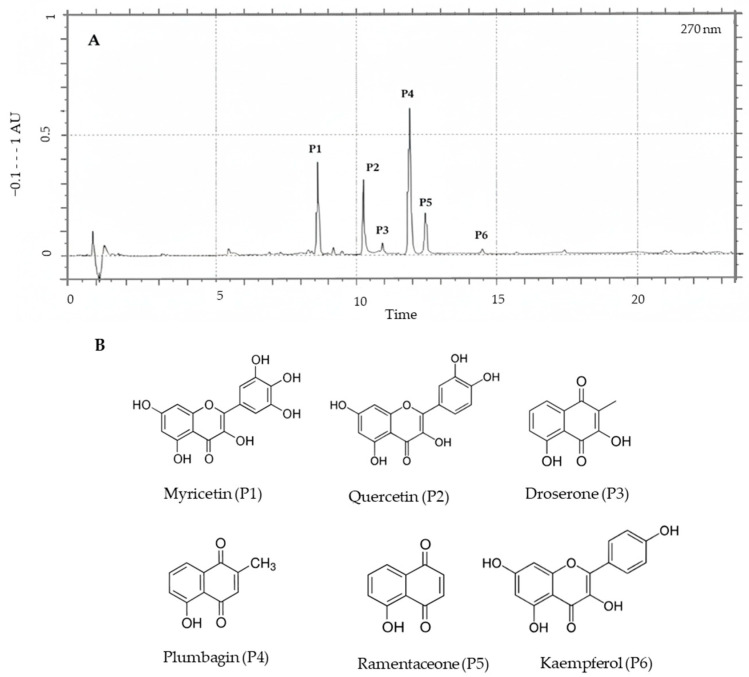
(**A**) HPLC of ethyl acetate extract from *Drosera intermedia* at 270 nm. P1, myricetin (**17**); P2, quercetin (**18**); P3, droserone (tentative identification); P4, plumbagin (**29**); P5, ramentaceone (**33**); P6, kaempferol (**19**). (**B**) The main compounds in the extract.

Based on the results obtained, the investigation was continued by analysing the chemical composition of the *D. intermedia* ethyl acetate extract to identify the active components in it. The chromatographic technique employed in this research was selected for its ability to provide excellent resolution, ensuring accurate identification and characterization of the compounds within the extract (Figure 3).

Individual phenolic compounds of ethyl acetate extract were identified by using HPLC-PAD and HPLC-ESI-MS/MS detection. The compounds identified in *D. intermedia* could be classified in naphthoquinones and flavonoids by their UV/vis spectra, and retention time as well as by their molecular ion and derived fragments obtained by ESI-MS in positive modes (Table 3).

Peaks P4 and P5 were identified as plumbagin (**29**, C_11_H_8_O_3_, 5-hydroxy-2-methylnaphthalene-1,4-dione) and ramentaceone (**33**, C_11_H_8_O_3_, 7-hydroxy-2-methylnaphthalene-1,4-dione), respectively. Both compounds exhibited identical UV spectra and mass spectra. Identification was performed by HPLC-PAD by comparing the retention time and UV spectrum with reference standards [51,52]. The chromatograms obtained for the two standards are shown in Figure S1 of the Supplementary Materials. Plumbagin (**29**) eluted at a t_R_ = 12.01 min, while ramentaceone (**33**) eluted at t_R_ = 12.42 min, with both showing a maximum absorbance (λ_max_ = at 266 and 414 nm). The chemical formula of both compounds was further confirmed by HPLC-MS. The detection of molecular ions and fragments by HPLC-MS allowed for the confirmation of their naphthoquinone structure. The protonated ion [M+H]^+^ at *m*/*z* 189.1 confirmed the molecular mass of both molecules. Subsequent fragmentations provided additional structural evidence. The ion at *m*/*z* 174.3 [M+H−CH_3_]^+^ corresponded to the loss of 15 Da from the protonated molecular ion. The appearance of the ion at *m*/*z* 161.5 [M+H−CO]^+^, resulting from a loss of 28 Da (attributable to a CO molecule), is a characteristic fragmentation pattern of naphthoquinone systems, reflecting the stability and reactivity of their carbonyl groups. Additionally, the ion at *m*/*z* 144.1 was formed by the consecutive loss of CO and H_2_O (45 Da from [M+H]^+^ or 17 Da from *m*/*z* 161.3), which confirms the presence of both quinone functionalities and the hydroxyl group within the molecule.

Peak P3 (t_R_ = 11.12 min) was tentatively identified as droserone (C_11_H_8_O_4_, 3,5-dihydroxy-2-methylnaphthalene-1,4-dione) based on its retention time, UV spectrum (269, 422 nm), and molecular ions. A comparison with a commercial standard could not be performed as none was available. HPLC-MS analysis in positive mode detected molecular and fragment ions reflecting its methylated dihydroxynaphthoquinone structure. A protonated molecular ion at *m*/*z* 205.0 ([M+H]^+^) confirmed the molecular mass of droserone. Additional ions were observed at *m*/*z* 190.0 ([M+H−CH_3_]^+^), which is characteristic of a methyl group loss (15 Da), and at *m*/*z* 177.0 ([M+H−CO]^+^), corresponding to a loss of carbon monoxide (28 Da) and characteristic of a quinone structure. The presence of hydroxyl groups (-OH) was established by the ion at *m*/*z* 187.0 ([M+H−H_2_O]^+^), corresponding to the loss of a water molecule (18 Da) from the protonated ion. A second ion at *m*/*z* 169 ([M+H−2H_2_O]^+^) further supported the presence of a second hydroxyl group, resulting from the loss of a second water molecule. The ion at *m*/*z* 162.0 ([M+H−CH_3_−CO]^+^) corresponds to the combined loss of a methyl group and a carbon monoxide molecule.

HPLC/DAD and HPLC/MS data indicated that peaks P1 (t_R_ = 8.87 min), P2 (t_R_ = 10.26 min), and P6 (t_R_ = 14.50 min) were flavonoids. All three peaks showed a typical flavonol UV-vis spectrum (λ_max_ = 254–265, 301sh, 374–365 nm) and molecular ions ([M+H]^+^) at *m*/*z* 319.4, 303.4, and 287.0, with a consistent mass difference of 16 Da between them, which demonstrates the presence of an additional hydroxyl (-OH) group in each successive compound. Peak P1 was identified as myricetin (**17**, C_15_H_10_O_8_, 3,5,7-trihydroxy-2-(3,4,5-trihydroxyphenyl)chromen-4-one), a hexahydroxyflavone with hydroxy groups at positions 3, 3’, 4’, 5, 5’, and 7. Peak P2 was identified as quercetin (**18**, C_15_H_10_O_7_, 3,5,7-trihydroxy-2-(3,4-dihydroxyphenyl)chromen-4-one), a pentahydroxyflavone with five hydroxy groups at positions 3, 3’, 4’, 5, and 7. Peak P6 was identified as kaempferol (**19**, C_15_H_10_O_6_, 3,5,7-trihydroxy-2-(4-hydroxyphenyl)chromen-4-one), a tetrahydroxyflavone with four hydroxy groups at positions 3, 5, 7, and 4’. These results are consistent with data published in publicly available databases [53,54,55].

These compounds have been previously described in several *Drosera* species [56,57,58,59]. A prior study demonstrated that the hexane extract of *D. intermedia* exhibited significantly higher antimicrobial activity than its methanol and water extracts [60]. HPLC-MS analysis of this hexane extract identified plumbagin as a single and high purity compound.

In contrast, the methanolic extract primarily contained flavonoids and only trace amounts of plumbagin, with the flavonoids likely contributing to its lower, residual activity [60]. These findings corroborate the present study, where both naphthoquinones and flavonoids were detected in the ethyl acetate extract. This is the first study to chemically characterize an ethyl acetate extract of *D. intermedia*, highlighting a novel aspect of this research.

### 2.4. Design of an Antifungal Formulation Using Drosera intermedia

Various antifungal formulations are used in postharvest applications to preserve fruit, prevent deterioration, and extend shelf life. These biocontrol agents are typically categorized by their mode of action. Edible coatings form a protective layer that reduces dehydration and delays ripening. Microorganisms, such as yeasts or bacteria, act as biocontrol agents by competing with pathogenic fungi for space and nutrients or by producing inhibitory substances. Finally, contact or protective fungicides prevent fungal spore germination and development and are commonly applied via immersion baths or spraying.

Synthetic contact fungicides have been the standard in the food industry for a long time. However, their use is declining due to growing concerns over residual chemical contamination and the increasing prevalence of fungal strains that are resistant to them. Consequently, the search for and implementation of natural antifungals has become a critical area of research.

Based on the chemical characterization and activity results, the next phase of this research was focused on developing and evaluating an innovative antifungal formulation. This formulation, derived from a *D. intermedia* ethyl acetate extract, was designed for fruit preservation. To standardize the extract for this formulation, its major components were first quantified using calibration curves. (Table 4).

The ethyl acetate extract showed a higher concentration of total naphthoquinones than flavonoids (2.8% vs. 1.40%). Plumbagin (**29**) was the most abundant compound, representing 15% of the extract (35% of total phenolics), followed by ramentaceone (**33**) at 0.94% (22.5% of total phenolics) and myricetin (**17**) at 0.69% (16.5% of phenolics). This naphthoquinone content gave the dried *D. intermedia* ethyl acetate extract a remarkable efficacy, achieving approximately 90% in vitro inhibition against the fungal pathogens at a concentration of 250 ppm.

The industry has developed a wide array of both synthetic and natural antifungal formulations for postharvest fruit preservation. These formulations are optimized for efficacy, stability, and ease of use, providing uniform coverage and prolonged action on the fruit’s surface. The selection of a specific formulation and application method is determined by the nature of the active ingredient, the crop, and the target pathogen. Spraying is generally considered the most suitable application method for a postharvest antifungal.

Emulsifiable concentrates (ECs) are among the most common commercial formulations for spraying. These liquid formulations contain the active ingredient dissolved in an organic solvent, along with an emulsion agent. Emulsifiers improve the solubility and stability of lipophilic compounds and once mixed with water, they form an emulsion. The resulting dispersed droplets provide excellent coverage and adhesion to the fruit’s surface superior to that of a simple aqueous solution [61]. Therefore, the formulation strategy was focused on developing an emulsifiable concentrate to be diluted with water for application. Table 5 details the composition of the designed formulation.

Based on the promising in vitro results—which showed approximately 90% inhibition at a concentration of 250 ppm—an emulsifiable concentrate was designed. The formulation was developed to ensure the ethyl acetate extract could disperse uniformly in water, creating a fine and effective spray for application. The final concentrate was formulated at a 50 mg/mL (5%) concentration.

A key obstacle to formulating the ethyl acetate extract is its inherent low water solubility, a direct consequence of its nonpolar characteristics. To overcome this, an emulsification strategy was used, dispersing the extract within an aqueous phase using surfactants. The developed formulation included 15% Polysorbate 80 (Tween^®^-80) as the primary emulsifier. This non-ionic surfactant has a high HLB value, which facilitates the stabilization of the emulsion and improves the dispersion of the active compound in the aqueous phase [62]. In addition to helping stabilize the emulsion, it improves the dispersion of the active ingredient in the aqueous phase. The formulation also included propylene glycol (12%) and glycerol (5%) as co-solvents and humectants. These not only contribute to the emulsion’s stability but can also improve the wetting and adherence of the spray on the fruit’s surface [63]. Xanthan gum (0.2%) served as a thickener and stabilizer, preventing particle sedimentation and ensuring a uniform application. It also contributed to reduce the surface tension between incompatible substances like oil and water, helping to form a stable emulsion [64]. Citric acid (0.2%) was added to adjust the formulation’s pH to 5.0, maximizing both antifungal activity and product stability. Ascorbic acid (0.1%) was included as an antioxidant to protect the bioactive compounds from degradation during storage and application, while also serving as a pH adjuster [65]. Finally, sodium benzoate (0.1%) was incorporated as a preservative to ensure the formulation’s long-term microbial stability [66].

The stability of the emulsifiable concentrate was evaluated over a 7-day period by performing a physicochemical characterization. Samples were stored at room temperature in the dark. The main physicochemical properties of the emulsifiable concentrate are summarized in Table 6.

The physicochemical characterization results confirm that the emulsifiable concentrate possesses the necessary properties for efficient and stable application, maintaining its structural and functional integrity for at least 7 days. The pH of the formulation remained stable (pH = 5.0). This acidic pH is ideal, as it often contributes to the stability of many emulsions and can be beneficial for antifungal activity in post-harvest environments. The consistent pH indicates that no significant hydrolysis reactions of the formulation’s components occurred. The droplet size, a critical parameter for both efficacy and stability, remained constant at approximately 1.02 µm. The Polydispersity Index (PDI), which measures the size distribution of the droplets, was maintained at low values (0.17–0.19). This indicates a homogeneous and narrow size distribution, suggesting resistance to coalescence and maintenance of the emulsion’s structure over time. The Z-potential is an indicator of the electrostatic charge of the particles within an emulsion. A high absolute value, in this case around −40 mV, signifies a strong electrostatic repulsion between the droplets. This repulsion prevents droplets from aggregating and coalescing, which is fundamental for the long-term stability of the formulation. The fact that this value remained relatively constant over the 7-day period is further proof of the emulsion’s robustness. Finally, the viscosity of the formulation remained stable, with a slight and non-significant increase from 3126 cP to 3140 cP. This relatively high viscosity contributes to physical stability by reducing the movement of the droplets, which in turn decreases the probability of collisions that could lead to coalescence. The stability of the viscosity is a strong indication of the formulation’s resistance to sedimentation or creaming, ensuring the product maintains its consistency and uniformity over time.

The formulation was tested at four different doses: 250, 500, 1000, and 2000 ppm. These concentrations correspond to 5–40 mL of the emulsifiable concentrate in 1 L of the final solution, equivalent to a final extract concentration of 0.025–2 mg /mL, or 0.025, 0.05, 0.1, and 0.2%. The results of this dose–response study and EC50 values are presented in Table 7 and Table 8. For comparative purposes, difenoconazole and the commercial systemic fungicide Neozil 50EC^®^, formulated with imazalil, was used as a positive control. All *p*_Tukey_ values are included in Table S1–S4 of Supplementary Materials. Imazalil belongs to the imidazole group of fungicides and acts by inhibiting the biosynthesis of ergosterol in the fungal cell membranes, which disrupts fungal growth and reproduction. Its systemic action allows it to penetrate the fruit’s skin, providing both preventive and curative action against incipient infections.

The results presented in Table 7 demonstrate a clear dose–response relationship in the inhibition of the tested fungi. The dose–response curves for the formulation (per pathogen) are shown in Figure S2 of the Supplementary Materials. The formulation exhibited minimal activity at a low concentration of 250 ppm (0.09% to 4% inhibition). A statistically significant increase in efficacy was observed at 500 ppm for all pathogens. For instance, inhibition of *Rhizopus stolonifer* increased to 50%, a highly significant difference from 250 ppm (*p*_Tukey_ = 0.005, Table S4 of Supplementary Materials). The formulation achieved a high level of efficacy at 1000 ppm, surpassing 65% inhibition in all cases. At this concentration, its performance was comparable to the reference fungicide Neozil 50EC^®^ (73% to 85%). Increasing the concentration to 2000 ppm did not lead to a substantial increase or statistically significant increase in inhibition for *A. alternata*, *P. expansum*, and *R. stolonifer*. dose. For *P. expansum*, the inhibition of 86% at 2000 ppm was not statistically different from that observed at 1000 ppm (65%) (*p*_Tukey_ = 0.790, Table S3 of Supplementary Materials). In contrast, *B. cinerea* showed a significant increase in inhibition at 2000 ppm (92%), differing significantly from the 1000 ppm dose (75%) (*p*_Tukey_ = 0.033, Table S2 of Supplementary Materials). This suggests a comparatively lower sensitivity to the formulation that necessitates a higher dose for optimal efficacy. The EC50 values (Table 8) further corroborate the observed potency differences. The formulation demonstrated the highest potency against *R. stolonifer* (500 ppm), a precise estimate supported by a narrow 95% confidence interval (458–618 ppm). This was followed by *P. expansum* (584 ppm) and *A. alternata* (595.3 ppm). The highest EC50 value was recorded for *B. cinerea* (645 ppm), confirming its greater resistance as a higher concentration is required to achieve the 50% inhibition threshold. At the 1000 ppm concentration, the performance of the formulation was comparable to the commercial standards in several instances. Against *P. expansum*, the formulation (65.1%) did noy show a statistically significant difference compared to Neozil 50EC^®^ (73%) (*p*_Tukey_ = 0.833, Table S3 of Supplementary Materials), or Difenoconazole (80%) (*p*_Tukey_ = 0.625). Against *B. cinerea*, the formulation at 1000 ppm (75%) was not significantly different from Neozil 50EC^®^ (79%) (*p*_Tukey_ = 0.974, Table S2 of Supplementary Materials). These results underscore that natural formulation achieves levels of activity comparable to systemic commercial fungicides against several strains, warranting its further development as a biofungicide alternative.

The antifungal activity of various plant extracts against the fungi studied here is extensively documented in the scientific literature [67,68,69,70]. However, few studies have progressed to the crucial step of developing a formulation for application. Recognizing that a substantial portion of disease control agents (25–30%) originate from natural sources [71], current research has increasingly focused on harnessing these compounds. In the field of postharvest management, plant extracts and essential oils, mainly, have been identified as promising alternatives to synthetic fungicides due to their potent antifungal properties [72]. For example, encapsulation of an alcoholic neem seed extract has been described for its efficacy against *Colletotrichum nymphaeae* and *Botrytis cinerea* [73]. Recent advances have focused on developing new, non-synthetic delivery systems. These include antifungal edible coatings based on citrus pectin and beeswax, enriched with eugenol, geraniol, propolis extract, or essential oils from *Satureja montana*, *Cinnamomum zeylanicum*, or *Commiphora myrrha*. These coatings were developed as effective alternatives to reduce sour rot and preserve the postharvest quality of ‘Valencia’ oranges against *Geotrichum citri-aurantii* [11]. Food-grade liquid lipid nanodroplets and solid lipid nanoparticles have also been used to encapsulate essential oils like cinnamaldehyde, eugenol, and thymol. These emulsions are subsequently incorporated into packaging systems to combat *R. stolonifer*, *A. alternata*, and *Aspergillus niger* [74]. A recent study provides a comprehensive review of the use of plant-derived antifungal agents and nanoencapsulation for postharvest pathogen management [75].

The promising results from *D. intermedia* extract suggest its potential for fruit preservation. However, its commercial application requires further essential trials. These include analyses of the emulsion’s stability, viscosity, and particle size to ensure consistent performance over several months under different storage conditions., as well as tests for adhesion and uniformity to evaluate surface coverage on fruit.

In the future, in vivo efficacy studies will be necessary to validate their protective effect under real-world conditions, which will be conducted following previously published experimental models [76]. Finally, compliance with food safety regulations will require toxicity studies, organoleptic evaluations to assess the extract’s impact on fruit taste and smell, and solvent residue analysis to ensure consumer safety.

## 3. Material and Methods

### 3.1. Chemicals and Reagents

All the solvents employed, analytical and HPLC grades, were purchased from Panreac (Barcelona, Spain). The detailed information for the standards used in this investigation, including the phytochemical family, commercial supplier, and catalog reference, is presented in Table S5 (Supplementary Materials). All reagents employed in this study were purchased from Sigma-Aldrich (MilliporeSigma, St. Louis, MO, USA), a brand of the Merck KGaA group (Darmstadt, Germany). Neozil^®^ 50EC was acquired from Laboratorios Agrochem S.L. (Esparreguera, Spain). Millipore-MilliQ distilled-deionised water (Millipore Ibérica S.A., Barcelona, Spain) was used throughout the experiments.

### 3.2. Plant Material

All dried and cut plant material was commercially acquired. The leaves of *Lawsonia inermis* L. (*Lythraceae*) were purchased from Plantarom, while the leaves of *Juglans regia* L. (*Juglandaceae*) and the whole plant of *Drosera intermedia* Hayne (*Droseraceae*) were obtained from Amorós Nature Technology.

### 3.3. Fungi Material

*Alternaria alternata* (Fiex ex Fries) Keissler 1912 (#2662), *Botrytis cinerea* (#2100), *Penicillium expansum* (#2275) and *Rhizopus stolonifer* (Ehrenberg: Fries) Lind. var. *stolonifer* (#2344) were obtained from “Colección Española de Cultivos Tipo” (Department of Microbiology, University of Valencia).

### 3.4. Plant Material Extraction

25 g of dry plant material was ground with liquid nitrogen and then macerated separately for 24 h at 4 °C using 500 mL of dichloromethane, ethyl acetate, or methanol. The extracts were centrifuged at 3500 rpm for 5 min. The clear supernatants were then collected, and the residues were re-extracted twice with fresh solvent. The supernatants from all three extractions were combined and concentrated by rotary vacuum evaporation at 40 °C. The resulting material was redissolved in water, lyophilized (Virtis BT3-SL, Gardiner, NY, USA), and stored in glass vial at −40 °C until further analysis. The extraction yields were calculated and expressed as percentage.

### 3.5. Chromatographic Analysis

#### 3.5.1. HPLC-PAD and HPLC-MS Analysis Instrumentation

The samples were analysed by HPLC-PAD using a Waters (Milford, MA, USA). The setup included a 600E multi-solvent delivery system, a Waters U6K sampler and a Waters 991 photodiode-array detector. Chromatography separation was performed on a C18 reversed-phase column (Nova-Pak, 150 mm × 3.9 mm, 4 µm, Waters, Milford, MA, USA) at 20 °C. The detection wavelength was monitored from 210 and 550 nm. The mobile phase consisted of a mixture of acetonitrile (A) and twice-distilled water adjusted to pH 3 with acetic acid (B, analytical grade) in different proportions. Elution was carried out at a flow rate of 1 mL/min using the following gradient program: 90% B from 0 to 1 min, decreasing to 70% B from 1 to 5 min, and further decreasing to 50% B from 5 to 25 min.

HPLC-MS analysis was performed using a HP 1100L liquid chromatograph coupled to a HP 100 MSD mass spectrometer with an API/electrospray interface (Agilent Technologies, Palo Alto, CA, USA). The column, mobile phase, time period and flow rate were similar to those used for HPLC-DAD analysis. The ESI interface was operated under the following conditions: gas temperature of 350 °C, nitrogen flow rate of 10 L/min, nebulizer pressure 24 psi, quadrupole temperature 80 °C and capillary voltage 3.5 kV. Nitrogen served as nebulizer and argon as collision gas. Mass spectra were acquired in both negative and positive ionization mode, with a scan range from *m*/*z* 50 to 500.

#### 3.5.2. Identification and Quantification of Individual Polyphenols

The identity of polyphenols was determined by combining data from HPLC-PAD and HPLC-MS analyses. Compounds were identified by comparing their retention times, UV-vis and mass spectra. Additionally, a co-injection with plumbagin and ramentaceone standards was performed to confirm the identity of the two isomeric naphthoquinones. Samples were dissolved in Methanol:Tetrahydrofuran (99:1, *v*/*v*). Standards were prepared at a concentration of 0.1 mg/mL. All solutions were filtered through a 0.45 mm polytetrafluorethylene (PTFE) filter before injecting 40 µL of each onto the system.

The HPLC-PAD method was validated for linearity, limit of detection, limit of quantitation precision, specificity, and accuracy according to the International Conference on Harmonization (ICH) guidelines. Individual polyphenolic compounds were quantified used a five-point regression curve based on authentic standards. Naphthoquinones were quantified using plumbagin as external standard (*y* = 2713*x* − 10.12, *r*^2^ *=* 0.998) while quercetin served as the standard of flavonoids (*y* = 2373.092*x* + 18.9, *r*^2^ *=* 0.997). The reproducibility of the chromatographic separations was verified on a second column, and a second HPLC system. Three samples were taken from each extract, and each sample was analysed three times by HPLC. For each extract, three independent samples were prepared, and each was analysed in triplicate. The content of each compound was expressed as mg per gram of freeze-dried extract.

### 3.6. Antifungal Activity

*A. alternata*, *B. cinerea*, *P. expansum* and *R. stolonifera* were used for the screening of antifungal activity of the products tested by using the method of mycelial growth [77]. The 32 standards and dichloromethane, ethyl acetate and methanol extracts from *L. inermis*, *J. regia* and *D. intermedia* were resuspended in sterile eppendorff with Tween 80 (0.1%) at concentration of 250 ppm and were added to sterile PDA medium. Difenoconazole was used as positive control.

The antifungal efficacy of the *D. intermedia* ethyl acetate extract was evaluated using a serial dilution to prepare concentrations of 250, 500, 1000, and 2000 ppm. For comparative analysis, two positive controls were run in parallel, difenoconazole solution at 250, 500, 1000 and 2000 ppm, and Neozil 50EC^®^ solution at 1000 ppm. Additionally, a negative control was prepared with a 0.1% Tween 80 solution to account for the potential antifungal activity of the solvent.

A 5 mm of fungi mycelium (taken from 7-day-old fungi culture) were inoculated in fresh medium containing Tween 80 (0.01%) (negative control), and in fresh medium containing Tween 80 plus sample. After incubation for 7 days at 25 °C in darkness, diameter of the growth zones was measured with Leica Q Win Program (v. 2.2) and converted into percentage of growth inhibition:

[(Control − Treatment)/Control] × 100.

The percentage is expressed as a mean of three replicate tests.

### 3.7. Antifungal Formulation

The antifungal formulation was prepared using a two-phase procedure. First, for the oily phase and surfactants, 5 g of *D. intermedia* ethyl acetate extract was dissolved in 12 g of propylene glycol. Both components were mixed with mild and constant agitation at 100 rpm to ensure the complete solubilization of the extract without introducing air into the mixture or generating unnecessary turbulence that could hinder the subsequent process. Then, 15 g of Polysorbate 80 (Tween^®^ 80) was added, and the solution was homogenized with constant stirring (100 rpm) at 45 °C for 15 min.

In a separate container for the aqueous phase, 62.4 mL of purified water was heated to 60 °C. The following components were then added one by one under constant stirring (200 rpm): 0.1 g of sodium benzoate (preservative), 0.2 g of citric acid (pH adjuster), and 0.1 g of ascorbic acid (antioxidant). It is important not to add a new ingredient until the previous one is completely dissolved. Once these ingredients were dissolved, 5.0 g of glycerol and 0.2 g of xanthan gum were added. The xanthan gum must be added slowly with vigorous agitation (600 rpm). Maintaining the temperature is important because heat increases the solubility of the xanthan gum and reduces the initial viscosity of the water, which facilitates its hydration, uniform dispersion, prevents the formation of clumps, and ensures a homogeneous solution.

Next, to form the emulsion, the oily phase was slowly added to the aqueous phase so that the oil particles would disperse uniformly in the water, facilitating the action of Polysorbate 80 as an emulsifier. During the entire emulsion formation process, constant stirring at 600 rpm was maintained at 60 °C. After the addition was complete, stirring was continued for 20 min to ensure the formation of a stable and uniform emulsion, reducing the risk of phase separation over time.

Finally, the formulation was then cooled to room temperature, and the final volume was adjusted to 100 g with purified water. The pH of the final formulation was measured and adjusted to 5.0 ± 0.2, and the resulting product was stored in a sealed container until use.

### 3.8. Characterization of the Physicochemical Properties of the Formulation

The physicochemical characterization of the emulsion was performed on days 0, 3, 5 and 7 days after preparation. Samples were stored at room temperature in the dark. The pH of the emulsion was measured with an Orion Versa Star Pro 52 pH meter (Thermo Scientific, Waltham, MA, USA). The hydrodynamic droplet size (z-average), polydispersity index (PDI), and Z-potential of the emulsion were determined by Dynamic Light Scattering (DLS) using a Zetasizer analyzer system (Brookhaven Instruments Corporation, Holtsville, NY, USA). The emulsifiable concentrate was diluted to a concentration of 0.1% (*v*/*v*) with filtered water (0.22 µm syringe filter). One millilitre of the diluted sample was then transferred to a disposable polystyrene cuvette. The measurement temperature was kept constant at 25.0 ± 0.1 °C with a detection angle of 173°. Finally, the viscosity of the emulsifiable concentrate was determined with an Alfa L rotational viscometer (Fungilab, Hauppauge, NY, USA).

### 3.9. Statistical Analysis

Each experimental condition was analysed with three independent biological replicates, with each replicate measured in triplicate (*n* = 9). Means and standard deviations were calculated using Microsoft Excel 2013 (Microsoft Corp., Redmond, WA, USA). Statistical analysis was performed using Stata v.12 (StataCorp LLC, College Station, TX, USA). Normality was verified using the Shapiro–Wilkinson test. Differences across groups for each pathogen were estimated using one-way ANOVA. Since ANOVA only indicates an overall difference, a post-hoc pairwise comparison test using Tukey’s method (95% CL) was subsequently performed to identify specific group differences. Tukey’s method served as the multiplicity handling technique, maintaining the global Type I error rate at α = 0.05 for all comparisons. Differences with *p* < 0.05 were considered statistically significant.

## 4. Conclusions

The present study reveals the significant antifungal potential of extracts from the carnivorous plant *Drosera intermedia*, identifying naphthoquinones as the primary active compounds responsible for their efficacy. In vitro results demonstrated that both the ethyl acetate extract and plumbagin, a key secondary metabolite, exhibit high inhibitory activity against fungal pathogens, specifically *Alternaria alternata*, *Rhizopus stolonifer*, *Penicillium expansum*, and *Botrytis cinerea*.

The development of an emulsifiable concentrate from the *D. intermedia* extract is not merely a technological advancement but is also directly aligned with the principles of sustainability. By utilizing a complex extract rich in naphthoquinones and flavonoids, our strategy supports the hypothesis that synergistic interactions among multiple natural compounds can be more effective and economically advantageous than synthetic alternatives. The formulation showed its optimal effect at 1000 ppm. This high efficacy positions it as a promising and viable alternative for managing rot, reducing the reliance on conventional synthetic chemicals.

While the in vitro efficacy is highly encouraging, the successful transition of this innovation to commercial use necessitates further research that also addresses sustainability. Future studies must meticulously optimize the dosage and validate in vivo efficacy, while ensuring the production and application processes minimize environmental impact. A rigorous demonstration of its food safety, including an absence of unacceptable residues and a lack of negative organoleptic impacts on the fruit, are indispensable for its acceptance in a market that increasingly prioritizes health and sustainability.

In conclusion, this research provides compelling evidence for the antifungal potential of natural products. These findings are particularly relevant in the context of rising resistance to synthetic fungicides and the urgent need for ecological alternatives. The results underscore the promise of naphthoquinones as candidates for the development of new antifungal agents, offering a sustainable solution with a novel mechanism of action to address the challenge of fungal infections and protect crops responsibly.

## Figures and Tables

**Table 1:** Antifungal activity, expressed in percentage, of phenolic compounds.

		Growth Inhibition (%)			
		*Alternaria alternata*	*Botrytis cinerea*	*Penicillium expansum*	*Rhizopus stolonifer*
Phenols	Phenol (**1**)	-	18 ± 1	4.5 ± 0.1	-
	Catechol (**2**)	-	29 ± 1	19 ± 2	9.0 ± 0.7
	Pyrogallol (**3**)	71 ± 4	66 ± 2	3.0 ± 0.1	-
Hydroxyphenolic acids	Benzoic acid (**4**)	0.6 ± 0.1	-	-	-
	*p*-hydroxybenzoic acid (**5**)	-	14 ± 3	-	-
	Protocatechuic acid (**6**)	-	28 ± 1	41 ± 2	10 ± 1
	Gallic acid (**7**)	12.1 ± 0.4	57 ± 3	0.4 ± 0.1	62 ± 5
Hydroxycinnamic acids	*p*-coumaric acid (**8**)	7.3 ± 0.7	2.0 ± 0.1	-	-
	Caffeic acid (**9**)	25 ± 3	5.2 ± 0.1	3.0 ± 0.3	-
	Ferulic acid (**10**)	21 ± 2	5.5 ± 0.2	-	-
Coumarins	Coumarin (**11**)	-	-	-	-
	Esculetin (**12**)	-	7 ± 1	10 ± 2	-
Flavones	Chrysin (**13**)	-	-	-	-
	Acacetin (**14**)	-	0.9 ± 0.1	-	-
	Apigenin (**15**)	-	28 ± 2	-	19 ± 2
	Luteolin (**16**)	-	28 ± 3	-	19 ± 3
Flavonols	Myricetin (**17**)	-	-	-	33 ± 2
	Quercetin (**18**)	-	-	-	16 ± 1
	Kaempferol (**19**)	-	-	-	-
Flavanones	Naringenin (**20**)	-	26 ± 2	-	14 ± 2
	Hesperetin (**21**)	-	10 ± 1	-	29 ± 3
Chalcones	Isoliquiritigenin (**22**)	39 ± 4	2.0 ± 0.1	25 ± 1	14 ± 2
Catechins	Catechin (**23**)	-	-	-	4.0 ± 0.4
	Epicatechin (**24**)	-	7.9 ± 0.9	-	1.6 ± 0.1
Benzoquinones	1,2-benzoquinone (**25**)	13 ± 1	-	16 ± 1	19 ± 2
	1,4-benzoquinone (**26**)	25 ± 4	15.2 ± 0.6	28 ± 4	30 ± 2
Naphthoquinones	Lawsone (**27**)	77 ± 5	86 ± 4	64 ± 1	80 ± 4
	Juglone (**28**)	31 ± 2	0.15 ± 0.01	16.4 ± 0.8	50 ± 4
	Plumbagin (**29**)	80 ± 1	65 ± 1	85 ± 3	86 ± 5
Anthraquinones	Emodin (**30**)	21 ± 2	49 ± 5	-	-
	Rhein (**31**)	36 ± 5	47 ± 4	10 ± 1	2.8 ± 0.5
	Anthraquinone (**32**)	11.0 ± 0.2	11 ± 2	-	-
Difenoconazole		78 ± 2	71 ± 2	80 ± 2	84 ± 2

Data are mean ± SD of three experiments performed in triplicates (*n* = 9). (-): No activity.

**Table 2:** Yield of extraction and antifungal activity of three medicinal plants with naphthoquinones.

		Yield of Extraction (%)	Growth Inhibition (%)			
Plant	Extract		*Alternaria alternata*	*Botrytis cinerea*	*Penicillium expansum*	*Rhizopus stolonifer*
*L. inermis*	Dichloromethane	4.26	-	-	-	-
	Ethyl acetate	8.51	71 ± 5	38 ± 4	95 ± 2	37 ± 2
	Methanol	20.00	29 ± 2	26 ± 1	31 ± 3	2.9 ± 0.1
*J. regia*	Dichloromethane	4.66	-	-	10 ± 1	19 ± 1
	Ethyl acetate	8.22	38 ± 2	-	47 ± 3	84 ± 3
	Methanol	13.44	19 ± 2	-	10 ± 1	18 ± 1
*D. intermedia*	Dichloromethane	5.44	2.1 ± 0.7	1.6 ± 0.6	38 ± 4	20 ± 2
	Ethyl acetate	10.09	87 ± 3	96 ± 3	64 ± 3	85 ± 4
	Methanol	12.60	21 ± 1	5 ± 1	22 ± 2	14.7 ± 0.9

Data are mean ± SD of three experiments (*n* = 3).

**Table 3:** Identification of the main compounds detected in *Drosera intermedia*.

Peak	Compound	t_R_ (min.)	Chemical Formula	Abs. Max UV (nm)	Molecular Weight	Mass Spectrum, *m*/*z* (RA%)
P1	Myricetin (**17**)	8.87	C_15_H_10_O_8_	254, 301sh, 374	318.2	319.4(100), 303.4(5), 287.0(30), 139.1(15), 121.2(10)
P2	Quercetin (**18**)	10.26	C_15_H_10_O_7_	254, 301sh, 368	302.2	303.4(100), 246.9(30), 267.0(30), 193.1(10), 175.0(20)
P3	Droserone *	11.12	C_11_H_8_O_4_	269, 422	204.2	205.0(100), 190.0(10), 187.0(30), 177.0(50), 172.0(10), 169.0(7), 162.0(22), 159.0(25)
P4	Plumbagin (**29**)	12.01	C_11_H_8_O_3_	266, 414	188.2	189.1(100), 174.3(58), 161.5(7), 144.1 (32)
P5	Ramentaceone (**33**)	12.42	C_11_H_8_O_3_	266, 415	188.2	189.0(100), 174.3(71), 161.3(12), 144.1 (25)
P6	Kaempferol (**19**)	14.50	C_15_H_10_O_6_	265, 301sh, 365	286.2	287.0(15), 251.4(100), 211.4(50), 165.2(30)

*: tentative identification.

**Table 4:** Quantification of main compounds of ethyl acetate extract from *Drosera intermedia*.

	Compound	mg/10 g Extract	g/100 g Dry Plant (%)
Flavonoids	Myricetin (**17**)	692 ± 27	0.69 ± 0.03
	Quercetin (**18**)	618 ± 22	0.62 ± 0.02
	Kaempferol (**19**)	95 ± 2	0.09 ± 0.02
	Σ Flavonoids	1404 ± 50	1.40 ± 0.05
Naphthoquinones	Droserone *	362 ± 5	0.36 ± 0.05
	Plumbagin (**29**)	1456 ± 112	1.5 ± 0.1
	Ramentaceone (**33**)	929 ± 20	0.94 ± 0.02
	Σ Naphthoquinones	2748 ± 137	2.8 ± 0.1

Data are mean ± SD of three experiments performed in triplicates (*n* = 9). *: tentative identification.

**Table 5:** Composition of an antifungal formulation with a dry extract of *Drosera intermedia*.

Component	Quantity (% *w*/*w*)	Function
Dry extract of *D. intermedia* (10:1, ethyl acetate)	5.0%	Active Ingredient
Polysorbate 80 (Tween^®^ 80)	15.0%	Emulsifier/Surfactant
Propylene glycol	12.0%	Co-solvent/Humectant
Glycerol	5.0%	Humectant/Stabilizer
Xanthan gum	0.2%	Suspending agent/Thickener
Citric acid	0.2%	pH adjuster
Ascorbic acid	0.1%	Antioxidant/Stabilizer
Sodium benzoate	0.1%	Preservative
Purified water	Q.s. 100%	Diluent

**Table 6:** Physicochemical Characterization of the Formulation.

	pH	Droplet Size (µm)	PDI	Z Potential (mV)	Viscosity (cP)
Day 0	5.0 ± 0.2	1.03 ± 0.01	0.17 ± 0.05	−41 ± 1	3126 ± 38
Day 3	5.0 ± 0.2	1.02 ± 0.03	0.17 ± 0.04	−41 ± 2	3134 ± 29
Day 5	5.0 ± 0.1	1.02 ± 0.04	0.19 ± 0.03	−400 ± 0.7	3135 ± 34
Day 7	5.0 ± 0.1	1.02 ± 0.03	0.19 ± 0.02	−40.0 ± 0.7	3140 ± 30

Data are mean ± SD of three experiments performed in triplicates (*n* = 9).

**Table 7:** Fungicidal activity of a *Drosera intermedia* formulation.

		Dose (ppm)	Growth Inhibition (%)			
			*Alternaria alternata*	*Botrytis cinerea*	*Penicillium expansum*	*Rhizopus. stolonifer*
Formulation		250	0.09 ± 0.01 ^a^	0.15 ± 0.01 ^a^	1.1 ± 0.2 ^a^	4 ± 1 ^a^
		500	41 ± 2 ^b^	25 ± 1 ^b^	46 ± 1 ^b^	50 ± 1 ^b^
		1000	80 ± 2 ^c^	75 ± 2 ^c^	65.1 ± 0.5 ^c^	85 ± 2 ^c^
		2000	81 ± 2 ^c^	92 ± 2 ^d^	86 ± 3 ^c^	88 ± 2 ^c^
Positive control	Neozil 50EC^®^	1000	82.0 ± 0.7 ^c^	79 ± 2 ^c^	73 ± 1 ^c^	85 ± 2 ^c^
	Difenoconazole	1000	78 ± 2 ^c^	71 ± 2 ^c^	80 ± 2 ^c^	84 ± 2 ^c^

Data are mean ± SD of three experiments performed in triplicates (*n* = 9). Differences were estimated by one-way ANOVA per pathogen followed by a post hoc pairwise comparison test using Tukey’s method (95% CL). In columns, different letters indicate values with significant differences (*p* < 0.05), and the same letters indicate values that do not show significant differences (*p* > 0.05).

**Table 8:** EC50 values and 95% IC ^a^ (in parentheses) of formulation against four pathogenic fungal species.

	*Alternaria alternata*	*Botrytis cinerea*	*Penicillium expansum*	*Rhizopus. stolonifer*
Formulation	595 ± 13 (560−766)	645 ± 14 (612−748)	584 ± 15 (455−624)	500 ± 7 (458−618)
Difenoconazole	503 ± 10 (482−573)	435 ± 9 (418−452)	471 ± 9 (453−519)	582 ± 11 (561−603)

Data are mean ± SD of three experiments performed in triplicates (*n* = 9). ^a^ 95% CI means 95% confidence Interval.

## Data Availability

The original contributions presented in this study are included in the article; further inquiries can be directed to the corresponding author.

## Supplementary Materials

The following supporting information can be downloaded at https://www.mdpi.com/article/10.3390/plants14223427/s1, Table S1. *p*_Tukey_-values of Fungicidal activity formulation against *Alternaria alternata*; Table S2. *p*_Tukey_-values of Fungicidal activity formulation against *Botrytis cinerea*; Table S3. *p*_Tukey_-values of Fungicidal activity formulation against *Penicillium expansum*; Table S4. *p*_Tukey_-values of Fungicidal activity formulation against *Rhizopus. stolonifer*; Table S5. Information of Authentic Standards; Figure S1. HPLC profile of (A) plumbagin (**29**) and (B) ramentaceone (B, **33**) standards; Figure S2: Dose–response curves for the formulation (per pathogen).
