# Supplementary material for: The Design of a Plant-Based Biopesticide Formulation with Extracts and Derivatives Containing Naphthoquinones
Source: Plants (Basel). 2025 Nov 9;14(22):3427. doi: 10.3390/plants14223427 (PMC12655655; doi:10.3390/plants14223427)
Supplement: Supplementary file 1 [file plants-14-03427-s001.zip › plants-3855040-supplementary.pdf]

Table S1. p_{Tukey}-values of Fungicidal activity formulation against *Alternaria alternata*

	F (250 ppm)	F (500 ppm)	F (1000 ppm)	F (2000 ppm)	Neozil 50EC® (1000 ppm)
F (500 ppm)	$p = 0.007$	-			
F (1000 ppm)	$p = 0.013$	$p = 0.028$	-		
F (2000 ppm)	$p = 0.031$	$p = 0.012$	$p = 0.501$	-	
Neozil 50EC® (1000 ppm)	$p = 0.019$	$p = 0.037$	$p = 0.223$	$p = 0.175$	-
Difenoconazole (1000 ppm)	$p = 0.001$	$p = 0.021$	$p = 0.218$	$p = 0.505$	$p = 0.340$

Differences were estimated by ANOVA followed by a post hoc pairwise comparison test using Tukey's method (95% CL). $p>0.05$: no significant differences $p<0.05$: significant differences

Table S2. p_{Tukey}-values of Fungicidal activity formulation against *Botrytis cinerea*

	F (250 ppm)	F (500 ppm)	F (1000 ppm)	F (2000 ppm)	Neozil 50EC® (1000 ppm)
F (500 ppm)	$p = 0.008$	-			
F (1000 ppm)	$p = 0.035$	$p = 0.027$	-		
F (2000 ppm)	$p = 0.009$	$p = 0.009$	$p = 0.033$	-	
Neozil 50EC® (1000 ppm)	$p = 0.012$	$p = 0.001$	$p = 0.974$	$p = 0.020$	-
Difenoconazole (1000 ppm)	$p = 0.003$	$p = 0.009$	$p = 0.041$	$p = 0.048$	$p = 0.420$

Differences were estimated by ANOVA followed by a post hoc pairwise comparison test using Tukey's method (95% CL). $p_{Tukey} > 0.05$: no significant differences $p_{Tukey} < 0.05$: significant differences

Table S3. p_{Tukey}-values of Fungicidal activity formulation against *Penicillium expansum*

	F (250 ppm)	F (500 ppm)	F (1000 ppm)	F (2000 ppm)	Neozil 50EC® (1000 ppm)
F (500 ppm)	$p = 0.007$	-			
F (1000 ppm)	$p = 0.024$	$p = 0.015$	-		
F (2000 ppm)	$p = 0.008$	$p = 0.009$	$p = 0.799$	-	
Neozil 50EC® (1000 ppm)	$p = 0.011$	$p = 0.008$	$p = 0.833$	$p = 0.964$	-
Difenoconazole (1000 ppm)	$p = 0.021$	$p = 0.015$	$p = 0.625$	$p = 0.751$	$p = 0.700$

Differences were estimated by ANOVA followed by a post hoc pairwise comparison test using Tukey's method (95% CL). $p_{Tukey} > 0.05$: no significant differences $p_{Tukey} < 0.05$: significant differences

Table S4. p_{Tukey}-values of Fungicidal activity formulation against *Rhizopus. stolonifer*

	F (250 ppm)	F (500 ppm)	F (1000 ppm)	F (2000 ppm)	Neozil 50EC® (1000 ppm)
F (500 ppm)	$p = 0.005$	-			
F (1000 ppm)	$p = 0.004$	$p = 0.041$	-		

F (2000 ppm)	$p = 0.008$	$p = 0.032$	$p = 0.519$	-	
Neozil 50EC® (1000 ppm)	$p = 0.004$	$p = 0.025$	$p = 0.636$	$p = 0.573$	-
Difenoconazole (1000 ppm)	$p = 0.007$	$p = 0.015$	$p = 0.391$	$p = 0.245$	$p = 0.687$

Differences were estimated by ANOVA followed by a post hoc pairwise comparison test using Tukey's method (95% CL). $p_{Tukey} > 0.05$: no significant differences $p_{Tukey} < 0.05$: significant differences

Table S5. Information of Authentic Standards

Phytochemical family	Compound	Commercial supplier	Catalog Reference
Phenols	Phenol (**1**)	Merck	W322318
	Catechol (**2**)	Extrasynthese	#0970
	Pyrogallol (**3**)	Merck	16040
Hydroxyphenolic acids	Benzoic acid (**4**)	Merck	242381
	p-hydroxybenzoic acid (**5**)	Extrasynthese	#6099
	Protocatechuic acid (**6**)	Extrasynthese	6050
	Gallic acid (**7**)	Extrasynthese	#4993 S
Hydroxycinnamic acids	*p*-coumaric acid (**8**)	Extrasynthese	#4751 S
	Caffeic acid (**9**)	Extrasynthese	#6034 S
	Ferulic acid (**10**)	Extrasynthese	#4753 S
Coumarins	Coumarin (**11**)	Extrasynthese	#0507 S
	Esculetin (**12**)	Extrasynthese	#0502
Flavones	Chrysin (**13**)	Extrasynthese	#1362 S
	Acacetin (**14**)	Extrasynthese	#1101 S
	Apigenin (**15**)	Extrasynthese	#1102 S
	Luteolin (**16**)	Extrasynthese	#1125 S
Flavonols	Myricetin (**17**)	Extrasynthese	#1127 S
	Quercetin (**18**)	Merck	PHL89262
	Kaempferol (**19**)	Extrasynthese	#1124 S
Flavanones	Naringenin (**20**)	Extrasynthese	#1128 S
	Hesperetin (**21**)	Merck	PHL89222
Chalcones	Isoliquiritigenin (**22**)	Extrasynthese	#1403 S
Catechins	Catechin (**23**)	Extrasynthese	#0976 S
	Epicatechin (**24**)	Extrasynthese	#0977 S
Benzoquinones	1,2-benzoquinone (**25**)	Cymit Química S.L.	4Z-B-126003
	1,4-benzoquinone (**26**)	Merck	PHR1028
Naphthoquinones	Lawsone (**27**)	Extrasynthese	#0424
	Juglone (**28**)	Merck	PHL80231
	Plumbagin (**29**)	Cymit Química S.L.	3B-P1139
	Ramentaceone (**33**)	Cymit Química S.L.	TM-T24705
Anthraquinones	Emodin (**30**)	Extrasynthese	#0453 S
	Rhein (**31**)	Extrasynthese	#0455 S
	Anthraquinone (**32**)	Merck	PHL82729

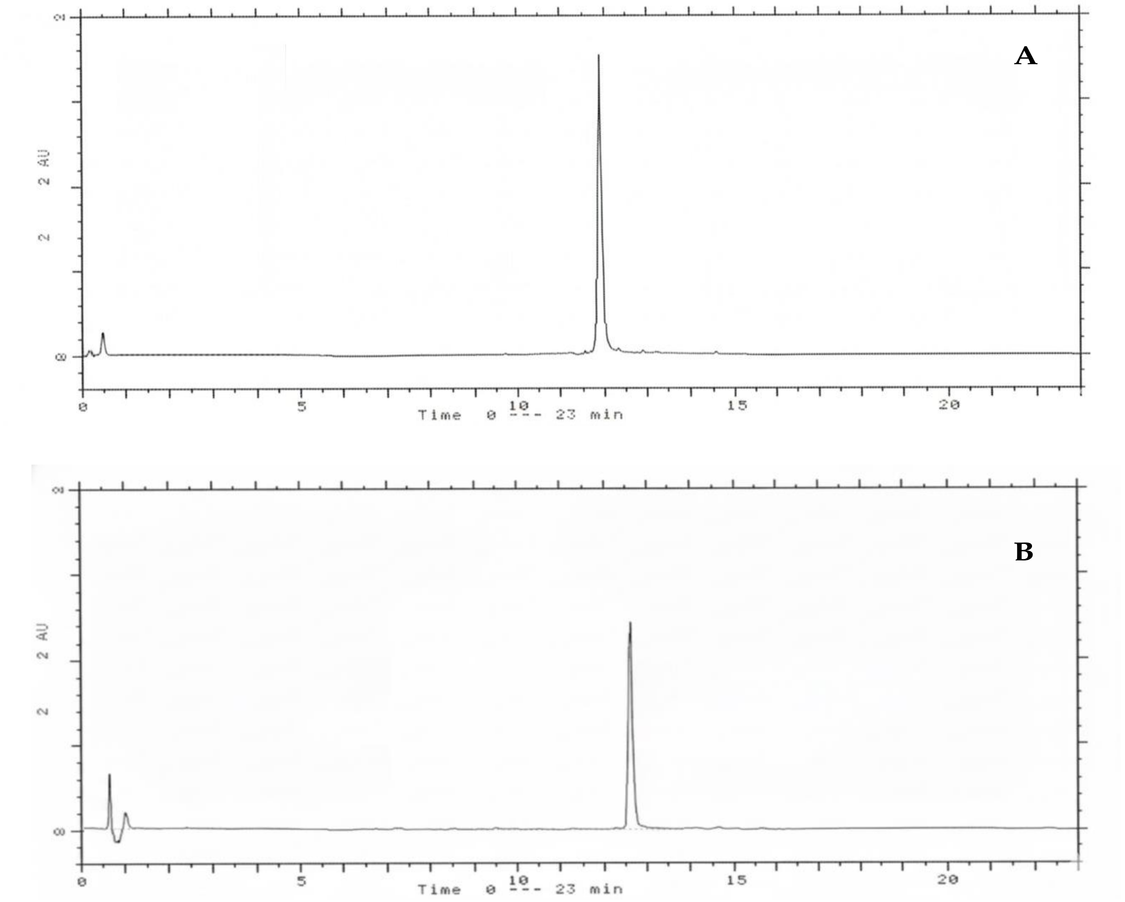

Figure S1. HPLC profile of (A) plumbagin (**29**) and (B) ramentaceone (B, **33**) standards.

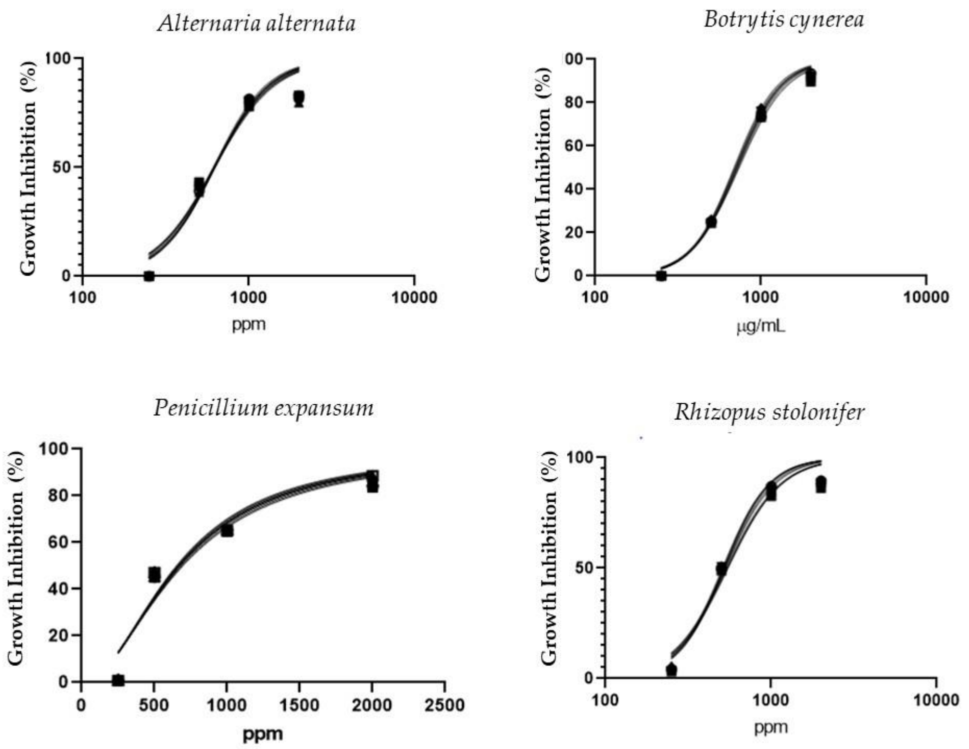

Figure S2. Dose–response curves for the formulation (per pathogen).
